# The principal mRNA nuclear export factor NXF1:NXT1 forms a symmetric binding platform that facilitates export of retroviral CTE-RNA

**DOI:** 10.1093/nar/gkv032

**Published:** 2015-01-27

**Authors:** Shintaro Aibara, Jun Katahira, Eugene Valkov, Murray Stewart

**Affiliations:** 1 MRC Laboratory of Molecular Biology, Francis Crick Avenue, Cambridge Biomedical Campus, Cambridge CB2 0QH, UK; 2 Biomolecular Networks Laboratories, Graduate School of Frontier Biosciences, Osaka University, 1-3 Yamadoka, Suita, Osaka 565-0871, Japan; 3 Department of Biochemistry, Graduate School of Medicine, Osaka University, 2-2 Yamadaoka Suita, Osaka 565-0871, Japan

## Abstract

The NXF1:NXT1 complex (also known as TAP:p15) is a general mRNA nuclear export factor that is conserved from yeast to humans. NXF1 is a modular protein constructed from four domains (RRM, LRR, NTF2-like and UBA domains). It is currently unclear how NXF1:NXT1 binds transcripts and whether there is higher organization of the NXF1 domains. We report here the 3.4 Å resolution crystal structure of the first three domains of human NXF1 together with NXT1 that has two copies of the complex in the asymmetric unit arranged to form an intimate domain-swapped dimer. In this dimer, the linkers between the NXF1 LRR and NTF2-like domains interact with NXT1, generating a 2-fold symmetric platform in which the RNA-binding RRM, LRR and NTF2-like domains are arranged on one face. In addition to bulk transcripts, NXF1:NXT1 also facilitates the export of unspliced retroviral genomic RNA from simple type-D retroviruses such as SRV-1 that contain a constitutive transport element (CTE), a *cis*-acting 2-fold symmetric RNA stem–loop motif. Complementary structural, biochemical and cellular techniques indicated that the formation of a symmetric RNA binding platform generated by dimerization of NXF1:NXT1 facilitates the recognition of CTE-RNA and promotes its nuclear export.

## INTRODUCTION

The export of mRNA from the nucleus through nuclear pores (NPCs) is the culmination of the nuclear phase of the eukaryotic gene expression pathway and releases mature mRNA transcripts into the cytoplasm for translation (reviewed by (1–3)). The movement of transcripts through NPCs is mediated by NXF1:NXT1 (also known as TAP:p15), which is the metazoan homolog of Mex67:Mtr2, the principal mRNA export factor in *Saccharomyces cerevisiae* (4,5). NXF1:NXT1 is functionally complementary to Mex67:Mtr2 and can rescue, at least partially, an otherwise inviable Mex67:Mtr2 deletion in *S. cerevisiae* (6). NXF1 is a modular protein comprised of four domains: an N-terminal RNA recognition motif (RRM), a leucine-rich repeat (LRR), a nuclear transport factor 2-like domain (NTF2L) and an ubiquitin-associated (UBA) domain (reviewed by (7)). NXT1/p15 is a 15-kDa protein that also has an NTF2-like fold and which forms a heterodimer with NXF1 via the NTF2L domain (3,7–10). Structures of the individual domains (11–13) have shown that both the NTF2L and UBA domain have hydrophobic surface depressions to which the phenylalanine–glycine (FG) motifs present in NPC proteins (nucleoporins or ‘nups’) can bind and this interaction is crucial in enabling the complex and its associated cargo to overcome the NPC barrier function (3,7). Efficient nuclear export of transcripts appears to require two independent nucleoporin binding domains and substitution of either the NTF2L or UBA for another NTF2L or UBA domain is capable of producing a partially functional NXF1 (14).

All of the nuclear steps of the gene expression pathway (including capping, splicing, cleavage and polyadenylation) need to have been completed before mature transcripts are exported to the cytoplasm for translation and these processes are coordinated by factors such as the TREX complex (15,16). The TREX complex associates with the spliceosome and is recruited to mRNA during splicing (16,17) or by specific RNA sequence motifs in the case of unspliced transcripts (18). Several adaptors, such as ALY and REF, that are components of the TREX complex, interact with the transcript before NXF1:NXT1 is finally loaded onto it to generate an export-competent mRNP (7,19–20). The molecular basis by which mature transcripts are recognized is unclear and appears to involve changes in RNA structure mediated by DEAD-box helicases (3). However, unspliced transcripts from simple retrovirus such as the Simian type-D retroviruses (including SRV-1 and MPMV) can circumvent the nuclear checkpoint mechanisms and be transported to the cytoplasm by utilizing the host NXF1:NXT1 machinery to export their RNA more efficiently than bulk mRNA. To do this, type-D retroviruses have a ∼130 nucleotide long RNA element known as the constitutive transport element (CTE) in the 3′ end of their single-stranded RNA genome (21,22) that binds to NXF1:NXT1 without the need for adapter proteins. The CTE-RNA is a 2-fold symmetric motif and both of its two symmetric lobes can bind NXF1:NXT1 independently (5). A CTE-like RNA motif has also been identified in an intron retained splice variant of the *NXF1* transcript and thus the CTE-mediated enhancement of mRNA export is a mechanism also utilized by metazoans natively (23).

Work conducted with *Xenopus* oocytes has identified the RRM and LRR domains of NXF1 to be the minimal requirement for binding viral CTE-RNA (24) and a crystal structure of NXF1^RRM-LRR^ bound to half of the CTE-RNA (CTE-B) shows an ‘L’-shaped CTE-B was bound by the two NXF1 domains. Phosphate-backbone interactions dominated for the RRM domain, whereas some based flipped interactions were employed in binding to the LRR domain. The two NXF1 domains also formed an ‘L’-shaped configuration, forming a ‘mutual molecular embrace’ (25). Although this crystal structure demonstrated how the RRM and LRR domains bind CTE-B, it remained unclear how the NXF1:NXT1 complex binds the full CTE-RNA. Moreover, it has recently been shown that the NTF2L domain also contributes to this interaction (26,27).

We present here the crystal structure of the complex formed between *Homo sapiens* NXF1 from which the N-terminal 95 residues and the UBA domain had been removed (NXF1^ΔNΔUBA^) and NXT1 and show that it has a novel homodimeric domain-swapped configuration generated by a interaction between the linker between the LRR and NTF2L domains with NXT1. The resultant domain-swapped dimer generates a flat platform with 2-fold symmetry in which the three pairs of RNA-binding domains (RRM, LRR and NTF2L domains) are all arranged on one face that results in their being ideally placed to interact with a dimeric binding motif such as that found in the CTE. Structure-guided mutagenesis of residues identified to be important components of the dimerization interface impact on the CTE-dependent enhancement of mRNA export, consistent with the formation of this dimeric platform contributing to the binding CTE-RNA. Intriguingly, these engineered mutations do not impair bulk mRNA export indicating that NXF1:NXT1 appears to bind CTE-RNA differently from bulk transcripts.

## MATERIALS AND METHODS

### Cloning and protein purification

PCR amplified *H. sapiens* NXF1^ΔNΔUBA^ (residues 96–555) and *H. sapiens* NXT1 were subcloned into the first and second multiple cloning site of the pETDuet-1 vector and co-expressed in *Escherichia coli* BL21-CodonPlus(DE3)-RIL cells (Agilent Technologies) by Isopropyl β-D-1-thiogalactopyranoside (IPTG) induction at 18°C for 16 h. Cells expressing NXF1^ΔNΔUBA^:NXT1 were lysed in 50 mM Tris–HCl pH 7.4, 500 mM NaCl, 20 mM imidazole pH 8.0. The lysate was clarified by centrifugation and the supernatant incubated with Ni-NTA beads for 1 h. The beads were pelleted by gravity flow filtration and washed with 60 ml of 50 mM Tris–HCl pH 7.4, 500 mM NaCl, 20 mM imidazole pH 8.0 after which the protein was eluted in 10 ml of the same buffer containing 250 mM imidazole pH 8.0. The His_6_ tag was removed by incubation overnight with TEV protease at 4°C. The NXF1^ΔNΔUBA^:NXT1 complex was purified to homogeneity by heparin affinity chromatography followed by gel filtration chromatography using a Superdex 200 column (GE Healthcare). Peak fractions containing pure stoichiometric NXF1^ΔNΔUBA^:NXT1 complex were pooled and concentrated and flash-frozen in liquid nitrogen and stored at −80°C in 20 mM Na–HEPES pH 8.0, 200 mM NaCl.

### RNA *in vitro* transcription and purification

CTE-RNA was produced by *in vitro* transcription. 0.04 mg/ml T7 RNA polymerase was incubated with 100 ng/μl DNA template, 7.5 mM NTPs, 50 mM MgCl_2_, 1 mM spermidine, 80 mM Na–HEPES pH 7.5 at 37°C for 4 h. Hammerhead (HH) ribozyme and Hepatitis Delta Virus (HDV) ribozyme were introduced into the 5′ and 3′ of the target to ensure a homogenous RNA product. Full-length CTE-RNA was separated from the two ribozymes by preparative urea–PAGE followed by gel extraction by electroelution using an EluTrap system (GE Healthcare). Eluted RNA was concentrated and buffer exchanged using a Millipore spin concentrator. RNA was stored until required at −20°C.

### Crystallography

Crystals of NXF1^ΔNΔUBA^ :NXT1 complex were grown at 18°C by sitting-drop vapor diffusion using 9% PEG 6000, 1 M LiCl, 0.1 M Na citrate buffer pH 5.1. Thin, blade-like crystals with *P1* symmetry were harvested and cryo-cooled in mother liquor supplemented with 20% glycerol. Crystallographic data was collected at the Diamond Light Source (Oxford, UK) using beamline I04-1. Reflections were indexed and integrated using XDS (28) and reflections merged and scaled using AIMLESS (29,30). An initial atomic model was obtained using molecular replacement in Phaser (31) using the individual domains from NXF1 as the search model (PDB ID: 1JKG and 3RW7). Iterative cycles of rebuilding building in COOT (32) and refinement in PHENIX (33) produced a final model with *R*_work­_/*R*_free_ of 21.3%/26.6% and excellent geometry (Table 1 and ref. 41).

**Table 1.** Crystal data

Symmetry	*P1*
Unit cell dimensions	
*a, b, c* (Å)	48.1, 84.0, 108
*α, β, γ* (°)	109, 99.8, 97.5
**Data collection**	
Wavelength (Å)	0.9200
Resolution range (Å)^a^	46.4–3.40 (3.67–3.40)
Total observations	77975
Unique observations	19892
Completeness (%)^a^	93.5 (94.0)
Multiplicity	3.9
*R*_pim_^a^	0.12 (0.49)
Mean *I* /*σ*(*I*)	6.5 (1.8)
**Refinement**	
*R*_work_/*R*_free_ (%)	23.1/26.6
Bond length rmsd (Å)	0.0043
Bond angle rmsd (°)	0.79
MolProbity score (41)/percentile	1.09/100
Ramachandran plot (%)	
Favored	98.5
Allowed	1.5
Forbidden	0

^a^Parentheses refer to final resolution shell.

### Small angle X-ray scattering

Small angle X-ray scattering (SAXS) was performed using an on-line HPLC system (Viscotek) equipped with Superdex 200 Increase 3.2/300 column (GE Healthcare) mounted on beamline BM29 at the European Synchrotron Radiation Facility (ESRF, Grenoble, France). Data collection was conducted at 20°C, using a wavelength of 0.995 Å and a sample-to-detector distance of 1 m. Data were processed automatically by the on-line pipeline AUTOSUB (part of the ATSAS package, (34)). Pair distance distribution functions of the particles *P*(*r*) and the maximum sizes *D*_max_ were computed using GNOM (35) and molecular weights were estimated by comparison of the extrapolated forward scattering *I*(0) of the samples obtained using Guinier analysis by AUTORG (34) with that of a bovine serum albumin standard (Sigma–Aldrich). *Ab initio* reconstructions were conducted using DAMMIF (36), without any symmetry constraints. After 10 DAMMIF runs, DAMAVER was used to analyze the normalized spatial discrepancy (NSD) between the 10 models and DAMFILT was used to generate an averaged model (37). Envelopes were calculated using SITUS pdb2vol based on the DAMFILT model (38).

### Isothermal titration calorimetry (ITC)

ITC measurements were conducted using an Auto-ITC_200_ calorimeter (Microcal). All proteins samples were buffer exchanged by dialysis into 20 mM Na–HEPES buffer pH 7.5, 200 mM KCl, 10 mM MgCl_2_. The RNA concentration in the injection syringe was 100 μM for CTE-RNA. The concentration of the NXF1^ΔNΔUBA^:NXT1 in the cell was 20 μM for the wild-type and 25 μM for the 3A mutant. The data were fitted using a one-site binding model using the Microcal ORIGIN software.

### Dual-luciferase reporter assay

HEK293F cells were cultured in Dulbecco's modified Eagle's medium supplemented with 10% fetal bovine serum. Transfection of plasmids was conducted as previously described (39). Luciferase reporter assays were performed using the dual luciferase reporter assay system according to the manufacturer's protocol (Promega). Three independent transfections were done for each plasmid.

### RNAi rescue assay

HEK293F cells were cultured in Dulbecco's modified Eagle's medium supplemented with 10% fetal bovine serum. Transfection of plasmids and siRNA were conducted as previously described, where the sequences of the siRNAs against dsRed and NXF1 are also given (39). Cells were visualized by confocal microscopy using signal from either GFP signal, Cy3 fluorescence from oligo-dT *in situ* hybridization (39) or Hoechst staining. Expression vectors coding for a siRNA resistant, GFP-tagged, wild-type and mutant NXF1 (GFP-NXF1^R^, GFP-NXF1^R-3A^, GFP-NXF1^R-3D^, GFP-NXF1^R-3D^) were constructed by introducing silent mutations to the siRNA target site (changing TCT ATC ATC ATC to agc ATt ATa ATt where lower case letters indicate the mutations introduced).

### Western blot analysis

Western blot analysis was conducted using standard methods using antibodies against NXF1 as previously described (6,39). Anti-GFP (Invitrogen), anti-GAPDH (Ambion) and anti-FLAG peptide (Sigma) antibodies were commercially acquired.

## RESULTS

### The 3.4 Å resolution crystal structure of NXF1^ΔNΔUBA^:NXT1 shows a novel domain-swapped dimer

**Figure 1.**
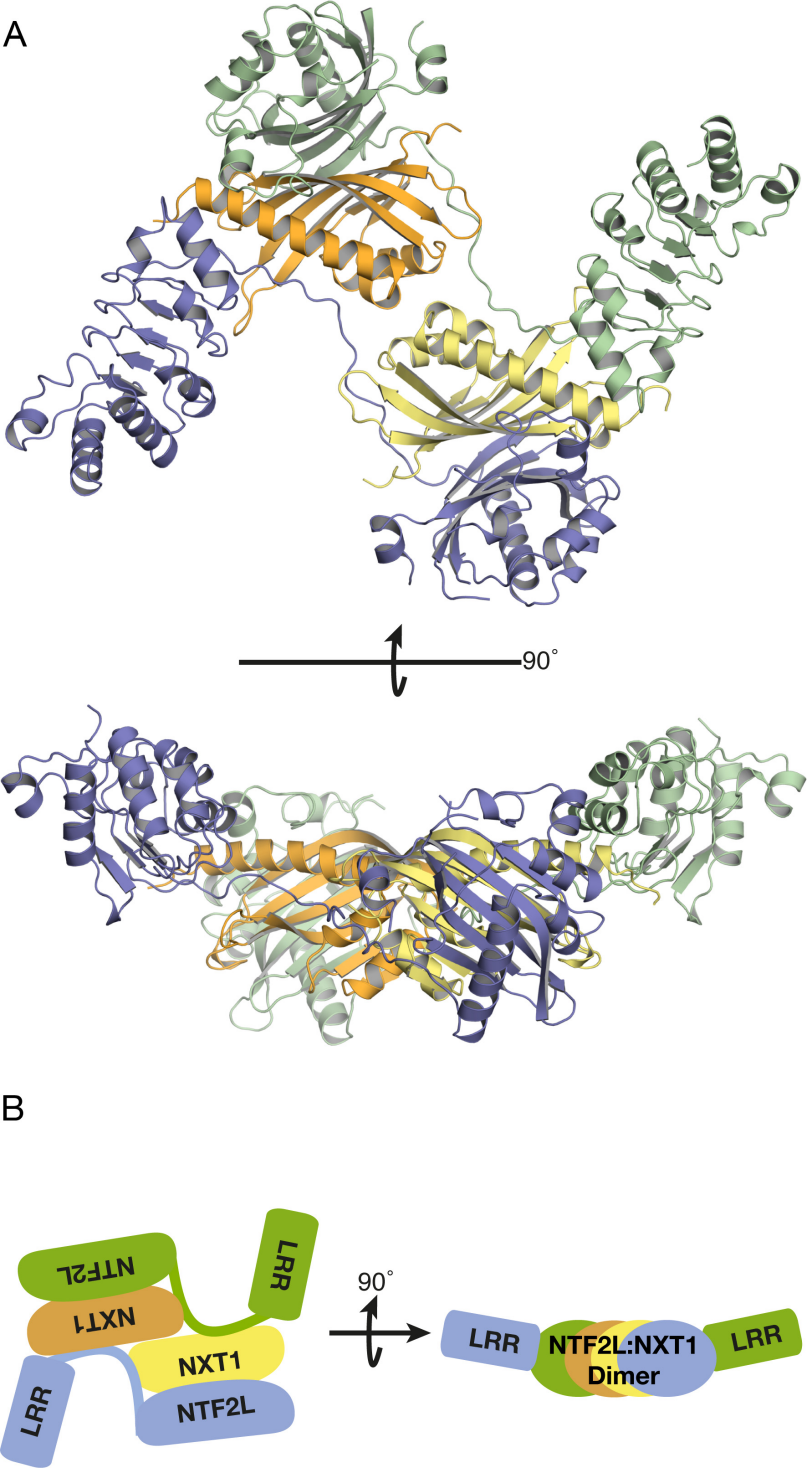
(**A**) Overview of the 3.4 Å resolution crystal structure of NXF1^ΔNΔUBA^:NXT1 and (**B**) Highly schematic illustration of the arrangement of domains in the domain-swapped dimer. Two copies of the protein were present in the asymmetric unit and continuous density was observed between the LRR and NTF2L domains. Density for the RRM domain was not observed, probably as a result of the flexibility of the linker between the RRM and LRR domain. The two copies of the protein in the asymmetric unit of the crystal formed a domain-swapped dimer where the domains were placed roughly in the same plane to form a flat platform with pseudo-2-fold symmetry.

**Figure 2.**
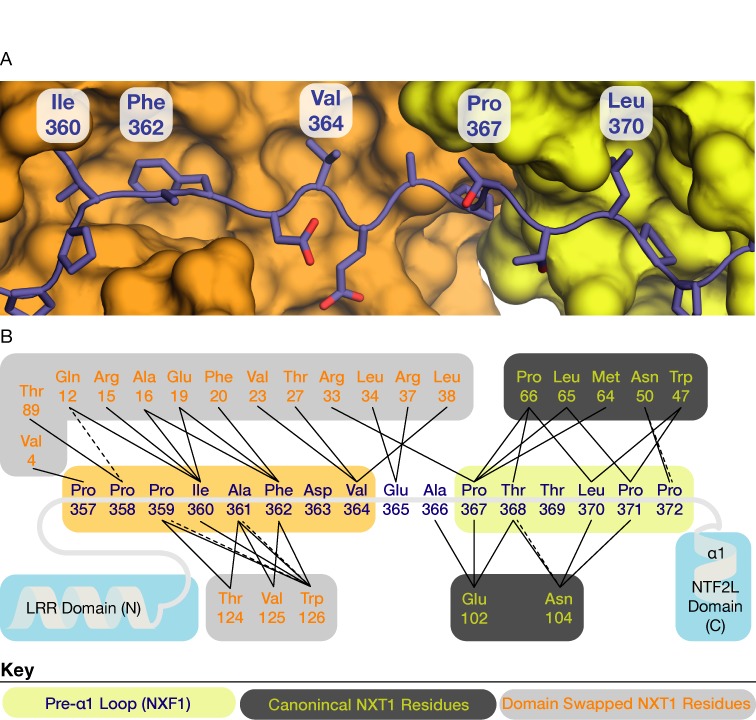
(**A**) Detailed view of the interaction between the linker between the LRR and NTF2L domains (blue) and the two copies of NXT1 (orange and yellow) in the asymmetric unit. Ile360, Phe362, and Val364 made extensive hydrophobic contacts with a domain-swapped NXT1 (orange) in a non-canonical manner. Interactions between the pre-α1 loop and the canonical copy of NXT1 (yellow) that was previously observed in the single domain structure (PDB ID: 1JKG) were also conserved in this multi-domain structure. (**B**) Schematic representation of the interactions between the LRR–NTF2L linker and the two copies of NXT1. Solid lines represent hydrophobic interactions and dotted lines represent putative hydrogen bonds. Key: pre-α1 loop residues (blue text on yellow); canonical NXT1 residues (yellow text on dark grey); domain-swapped NXT1 residues (orange text on light gray).

Because the linker between the C-terminal UBA domain and the remainder of NXF1 is thought to be very flexible (7) and the N-terminal 95 residues of NXF1 are thought to have an auto-inhibitory role in mRNA binding (40), these two regions were deleted to facilitate crystallization and analysis of RNA binding. A 3.4 Å resolution structure of NXF1^ΔNΔUBA^:NXT1 was obtained from *P*1 symmetry crystals by molecular replacement using the structures of the LRR and NTF2L:NXT1 fragments (PDB IDs: 3RW7 and 1JKG, respectively) as search models. The asymmetric unit contained two copies of the complex that were arranged to form a domain-swapped dimer (Figure 1). The structure was refined to an *R*_work_/*R*_free_ = 21.3/26.6% with excellent geometry (Table 1). Density for the RRM domain was not observed, probably as a result of its not having a well-defined position as a consequence of the flexibility of the RRM–LRR linker. Strikingly, the position of the LRR domain relative to the NTF2L:NXT1 region was the same in both copies. An interaction between the pre-α1 loop (residues 367–372) of the NTF2L domain, which had been observed previously in both the single domain structure of NXF1^NTF2L^:NXT1 (PDB ID: 1JKG) and the multi-domain structure of *S. cerevisiae* Mex67^ΔUBA^:Mtr2 (PDB ID: 4WWU), was conserved in the present multi-domain structure. The interaction between the pre-α1 loop and the canonical copy of NXT1 was largely dominated by hydrophobic interactions centering around residue Leu370 of NXF1 with NXT1 (mutated to Ala in the single domain structure by (12)) that dictated the spatial positioning of the LRR domain (Figure 2B, blue on yellow).

In addition to the interaction between the pre-α1 loop with the canonical NXT1 copy, the linker between the LRR and NTF2L domain also made contacts with the alternate non-canonical copy of NXT1 in a domain swapped manner (Figure 2B, blue on orange). Hydrophobic contacts also dominated this interaction interface, where Ile360, Phe362 and Val364 were buried in a cavity of the non-canonical copy of NXT1 (Figure 2A). Because Ile360, Phe362 and Val364 formed contacts principally with the non-canonical copy of NXT1, these three residues were attractive candidates for mutagenesis that would disrupt the dimeric platform via the non-canonical copy of NXT1 without disrupting the interaction between NXF1 and the canonical copy of NXT1. The pseudo-2-fold symmetric nature of this platform displayed by this crystal structure suggested that it might contribute to generating an interface for binding 2-fold symmetric RNA substrates such as the CTE-RNA and that formation of such a NXF1:NXT1 dimer could facilitate efficient nuclear export.

### The domain-swapped dimer generates a platform ideally suited for binding CTE-RNA

**Figure 3.**
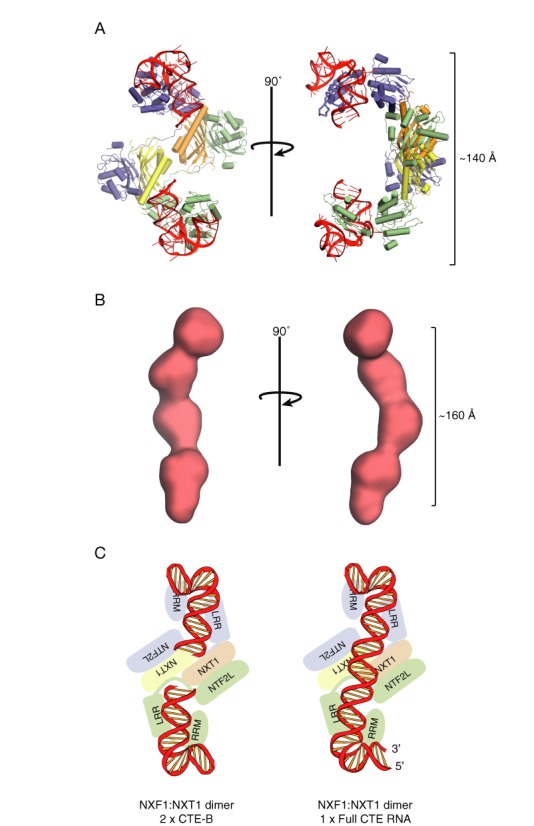
(**A**) Structural model in which two copies of NXF1^RRM-LRR^:CTE-B (PDB ID: 3RW6) were superimposed onto the two LRR domain present in the dimeric structure. The two ends of the stem loop of CTE-B face each other. (**B**) The *ab initio* reconstruction obtained from SAXS data of *in vitro* transcribed full-length CTE-RNA. The overall length of the full-length CTE-RNA was consistent with an elongated stem loop compatible with a length of 160 Å that is comparable with the model proposed in panel A in which the RNA was approximately 140 Å long. (**C**) Schematic representation of the model shown in panel A where the two CTE-B modules are orientated in a 2-fold symmetric manner. This arrangement of the two CTE-B roughly correspond to a full-length CTE-RNA which has 2-fold symmetry in the center that could span across the NXF1:NXT1 dimer.

Structural alignment using the LRR domains of two copies of the complex between a half CTE (CTE-B) and the NXF1 RRM and LRR domains ((25); PDB ID: 3RW6) with the current structure produced a plausible model for the way in which full-length CTE could be bound to NXF1:NXT1 (Figure 3A). The symmetric platform formed by the NTF2L:NXT1 regions in the domain-swapped dimer placed the two LRR domains in a spatial arrangement compatible for binding 2-fold symmetric substrates such as the CTE-RNA and which avoided steric clashes. The two copies of CTE-B were placed so that the ends of its stem loop faced each other so that the model corresponded approximately the predicted structure of the full CTE-RNA, in which a center of 2-fold symmetry exists around an AU-rich region in the center (21). Although in full CTE-RNA, one side of the RNA (CTE-A) would include the 5′ and 3′ end of the whole CTE-RNA stem loop (Figure 3C), these regions are not thought to be critical for the interaction (5) and so were omitted from the model. The resultant NXF1:NXT1:CTE-RNA model was about 140 Å long. To test if this dimension was reasonable, SAXS data was obtained for *in vitro* transcribed CTE-RNA and used to calculate a low-resolution envelope of the solution state of the molecule. *Ab initio* reconstructions using the DAMMIF package (36) produced an elongated envelope that contained five bulges (Figure 3B) that roughly corresponded to the non-base paired regions of the CTE-RNA, some of which are crucial for its binding to NXF1 (5). The envelope generated by SAXS was about 160 Å long and was consistent with the length of the NXF1:NXT1 dimer measured across the platform (Figure 3B).

**Figure 4.**
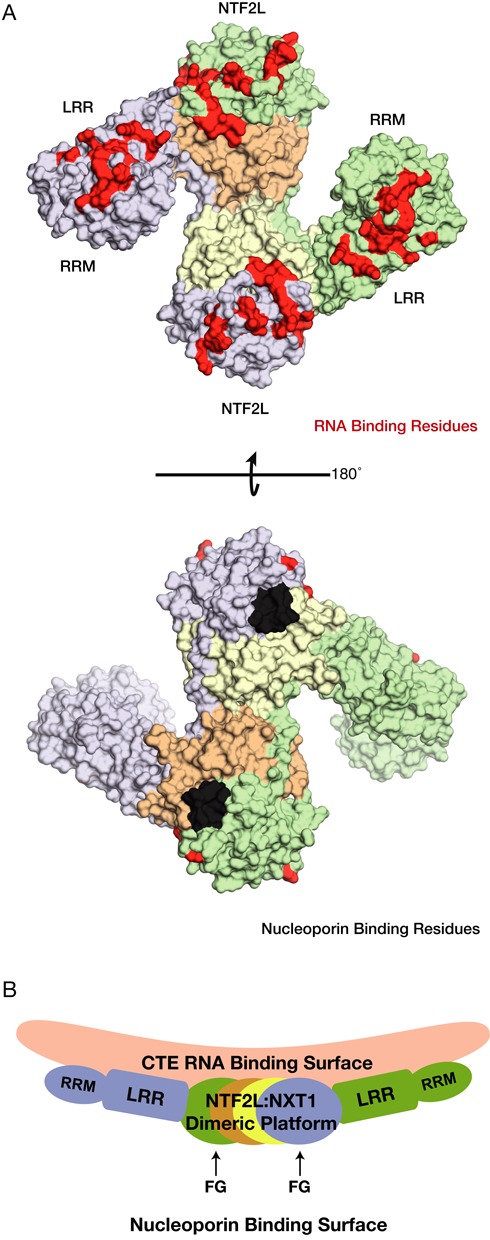
(**A**) Surface representation of the protein component from the model shown in Figure 3A. Residues implicated in RNA binding have been colored in red, whereas residues involved in contacts with nucleoporins have been colored in black. Residues not implicated in either RNA or nucleoporin binding have been colored according to the schematics used in Figure 3C. (**B**) Side view of the schematic of the model proposed for CTE-RNA binding. Importantly the FG nucleoporin binding sites are placed in a structurally compatible location with respect to the proposed RNA binding surface.

A striking feature of the model generated from the domain-swapped dimer was that the RNA-binding regions of the RRM, LRR and NTF2L domains were all arranged on the same face, opposite the regions that facilitate passage of the complex through the nuclear pores through interactions with the FG-nucleoporins that line the transport channel. Residues previously identified to form contacts with CTE-B in the RRM (residues 123, 125, 126, 128, 151–154, 156, 158, 190, 192) and LRR domains (residues 233, 248, 276, 278, 279, 304, 305, 307) were found clustered on one face of the NXF1:NXT1 dimer (25). Moreover, residues 429–469 that are located in the NTF2L domain and which are important in CTE-RNA binding (26) were also present on this face of the dimer (Figure 4A). Alanine substitution of charged residues in this region of the NTF2L domain dramatically reduced the binding of NXF1:NXT1 to half-CTE *in vitro* and also reduced the CTE-mediated enhancement of transcript export in dual-luciferase assays (accompanying manuscript). For simplicity, the region of the CTE linking the two NXF1-binding regions illustrated in Figure 3C was modeled as an ideal double helix, whereas structural predictions (21–23) indicate that this portion of the CTE-RNA probably contains some bulges and these could possibly enable it to be distorted slightly to facilitate reaching the putative binding sites on the NTF2L domain (see Figure 4A). Consistent with the requirement that the nucleoporin binding sites of NXF1:NXT1 need to be available while mRNA cargo is bound, the putative nucleoporin binding sites consisting of the residues 486–491 and 519–521 and 527 of NXF1 were found on the opposite face of the NTF2L:NXT1 dimer (12). Indeed in the NTF2L:NXT1 domain-swapped dimer, two nucleoporin binding sites from the NTF2L domain are brought into close proximity to one another, which could possibly facilitate the interactions between NXF1 and NPCs that mediate export. In summary, by locating the three RNA binding domains of NXF1 (RRM, LRR and NTF2L) on one face of the platform formed by the NTF2L:NXT1 domain-swapped dimer while placing two nucleoporin binding sites on the opposite face (Figure 4B) the proposed model provides a plausible structural basis for accommodating the simultaneous binding of CTE-RNA and nucleoporins needed for export through NPCs.

### Mutations in the LRR–NTF2L linker region reduced affinity for CTE-RNA and generated export defects

**Figure 5.**
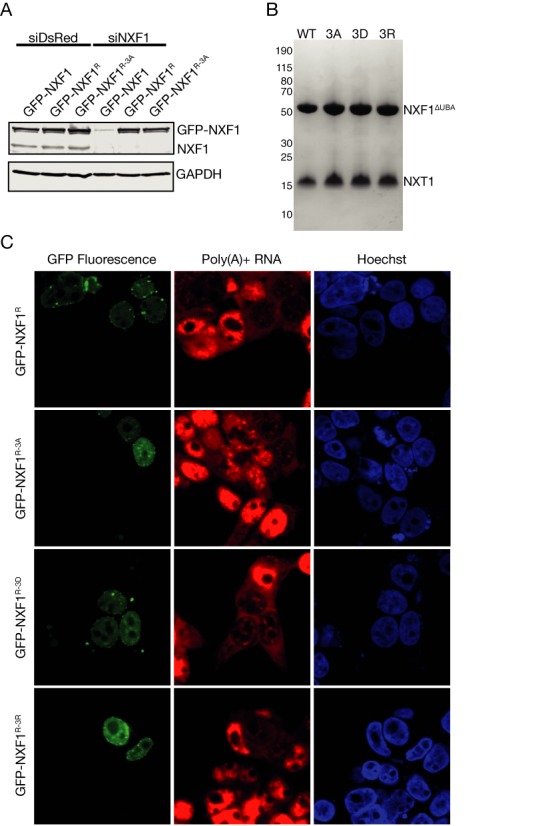
(**A**) Western blot analysis demonstrating the effective knock-down of NXF1 by siRNA silencing. The GFP-NXF1^R-3A^, GFP-NXF1^R-3A^ and GFP-NXF1^R-3A^ mutants had a silent mutation that resisted siRNA-based degradation. (**B**) Recombinant expression and purification of the mutant NXF1 constructs demonstrate that the NXT1 binding ability of the three mutant NXF1 was not altered. (**C**) Confocal microscopy demonstrates that cells expressing the three GFP-labeled NXF1 mutants that contained a silent mutation to resist siRNA-based degradation (GFP-NXF1^R-3A^, GFP-NXF1^R-3A^ and GFP-NXF1^R-3A^) were able to rescue the accumulation of poly(A)+ RNA in the nucleus, whereas cells in which GFP fluorescence was not present showed nuclear accumulation of poly(A)+ RNA.

To investigate the influence of platform formation of RNA nuclear export, the hydrophobic residues lining the non-canonical NXT1 hydrophobic pocket (Ile360 + Phe362 + Val364) were mutated to either alanine, aspartic acid or arginine (NXF1^3A^, NXF1^3D^, NXF1^3R^). These mutations were in regions not thought to be important with making direct contacts with RNA and so any impact they had on binding should instead reflect their altering the formation of the domain-swapped dimer. The expression levels of NXF1 mutants in HEK293F cells were comparable to that of wild-type and recombinantly purified protein from *E. coli* showed the ability to bind NXT1 was unaffected (Figure 5A and B). Crucially, the NXF1 mutants retained the ability to mediate the nuclear export of poly(A)+ mRNA in HEK293F cells. Because siRNA knockdown of wild-type NXF1 causes the accumulation of poly(A)+ RNA within the nuclei of HEK293F cells (39), it was possible to assay extent to which the mutant NXF1:NXT1 complexes could rescue the mRNA export block by expressing GFP-fused NXF1 constructs that contained a silent mutation (denoted by the superscripted ‘R’, Figure 5A) that enabled them to resist siRNA-based degradation. Western blot analysis demonstrated that in the presence of the siRNA (siNXF1), only the resistant GFP-tagged NXF1 constructs were present in the lysate, whereas when a control siRNA (siDsRed) was used both endogenous and expressed NXF1 could be detected (Figure 5A). Thus when endogenous NXF1 was depleted in siRNA knockdown experiments, expression of GFP-tagged mutant NXF1 was able to rescue the nuclear poly(A)+ RNA accumulation caused by the depletion of wild-type NXF1 (Figure 5C). The cellular localization of the NXF1 proteins containing mutations in the linker region also appeared to be unaltered and they were all observed to be located primarily in the nucleus (Figure 5C).

**Figure 6.**
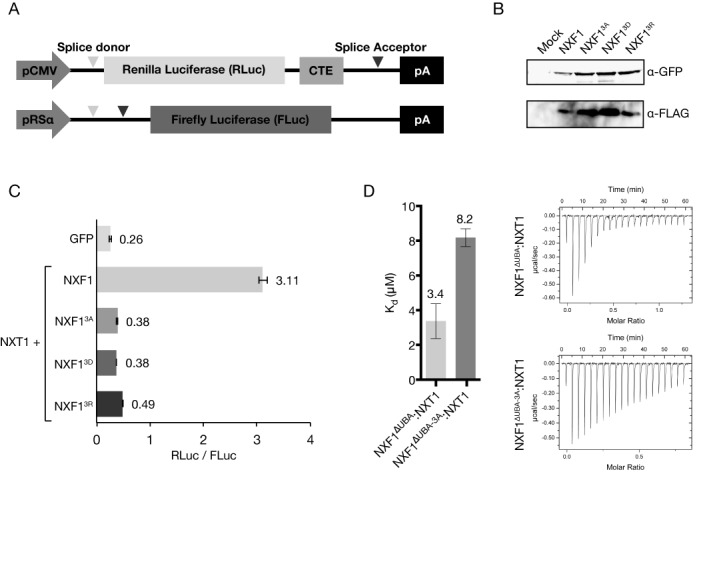
(**A**) Schematic of the two plasmids used in the modified dual luciferase reporter assay. The gene for RLuc is fused with the CTE, and thus in the presence of functional NXF1:NXT1 is expressed at higher levels than FLuc. (**B**) Western blot analysis confirmed that the triple mutations in the LRR–NTF2L linker did not alter levels of expression in HEK293F cells. Probing for FLAG-NXT1 also confirmed that all three NXF1 mutants (GFP-NXF1^R-3A^, GFP-NXF1^R-3A^ and GFP-NXF1^R-3A^) retained binding to NXT1. (**C**) Although wild-type NXF1 generated a 12-fold increase in the RLuc signal relative to that seen with a GFP control, all three NXF1 mutants tested showed greatly reduced enhancement of the RLuc signal. (**D**) ITC also showed that the triple alanine mutant (NXF1^3A^) has a ∼2.5-fold reduction in binding affinity to full CTE-RNA compared to the wild-type protein.

The impact of these NXF1 mutants on the nuclear export of CTE-RNA was assessed using a luciferase-based assay in which the two luciferase genes were co-transfected into HEK293F cells. The gene for *Renillia* luciferase (RLuc) was fused with the CTE coding sequence and, in the presence of functional NXF1:NXT1, the transcript for RLuc would be exported preferentially over *Firefly* luciferase (FLuc) (Schematically shown in Figure 6A). The ratio of RLuc signal over FLuc signal was interpreted to be related to the amount of transcript exported from the nucleus. When wild-type NXF1:NXT1 was overexpressed, the RLuc activity observed increased 12-fold relative to a GFP control. In contrast, with all three mutants (NXF1^3A^, NXF1^3D^, NXF1^3R^), the enhancement RLuc activity observed relative to GFP was less than a factor of 2 (Figure 6C). All three NXF1 mutants tested had expression levels comparable to the wild-type protein as demonstrated by western blot analysis (Figure 6B). ITC measurements indicated that the NXF1^ΔNΔUBA+3A^:NXT1 mutant bound CTE-RNA ∼2.5-fold more weakly than the wild-type protein (Figure 6D).

## DISCUSSION

### Dimerization of NXF1:NXT1 generates a RNA binding platform for CTE-RNA

The crystal structure of the NXF1:NXT1 complex indicated that it could adopt a novel dimeric configuration whereby a platform-like structure was generated as a result of the NTF2L:NXT1 region making domain-swapped interactions with the LRR domain. A defined spatial arrangement of the LRR domain relative to the NTF2L:NXT1 was also observed in both copies present in the asymmetric unit and this suggested a potential mechanism for NXF1:NXT1 binding symmetric RNA substrates such as the CTE-RNA motif. Hydrophobic residues present in the LRR–NTF2L linker followed by the pre-α1 loop of the NTF2L domain contributed to the formation of a continuous interaction interface with both copies of NXT1 that corresponded to the interaction with the canonical NXT1 previously observed in the single domain structure of NXF1^NTF2L^:NXT1 (12). Previously unrecognized contacts between the LRR–NTF2L linker region and a non-canonical copy of NXT1 appeared to be critical for dimer formation. Mutation of key hydrophobic residues in this region impacted on CTE-mediated RNA export as assessed *ex vivo* using dual-luciferase assays.

Structural alignment using two copies of the complex between a half CTE (CTE-B) and the NXF1 RRM and LRR domains (25) indicated that the CTE-RNA would be placed on the opposite side to the FG–nucleoporin binding site (12). This would be consistent with maintaining a separate unhindered hydrophobic surface that could interact with nuclear pore proteins and a hydrophilic positively-charged surface that could bind RNA cargo. One effect of forming a dimeric platform in such a way would imply that there would be a total of four nucleoporin binding sites in close proximity. By virtue of avidity, an increased number of nucleoporin binding sites may facilitate the rapid export of CTE-containing transcripts.

It has been previously reported that mutations in *S. cerevisiae* Mtr2 (E106G and R109G) disrupt pre-60S ribosomal subunit export but not bulk poly(A)+ RNA export. The observation that the disruption of NXF1:NXT1 dimerization inhibits CTE-mediated export, but not bulk poly(A)+ RNA may be an analogous situation in metazoans. The positioning of the LRR domain with respect to the NTF2L domain may be important for the export of a subset of highly structured RNAs such as the 5S rRNA in *S. cerevisiae* or the CTE-containing RNA for primates.

In summary, the 3.4 Å resolution crystal structure of NXF1:NXT1 has shown that this complex has the potential to form a novel domain-swapped dimer conformation that depends critically on interactions involving the pre-α1 loop and which generates a potential interaction platform in which all of the regions of the three RNA-binding domains of NXF1:NXT1 are located on one face. This arrangement is ideally suited to binding dimeric RNA motifs such as that of CTE-RNA and mutations engineered to impact on dimer formation reduce the nuclear export of CTE-RNA *in vivo*.

## ACCESSION NUMBER

Coordinates and structure factors for the 3.4 Å resolution crystal structure of NXF1:NXT1 have been deposited in the Protein Data Bank with accession number: 4WYK.
